# Maximising Triathlon Health and Performance: The State of the Art

**DOI:** 10.3390/sports13030066

**Published:** 2025-02-21

**Authors:** Veronica Vleck, Maria Francesca Piacentini

**Affiliations:** 1 CIPER, Faculdade de Motricidade Humana, Universidade de Lisboa, Cruz Quebrada, 1499-002 Lisbon, Portugal; 2 Department of Movement, Human and Health Sciences, University of Rome ‘Foro Italico’, 00135 Rome, Italy; mariafrancesca.piacentini@uniroma4.it

It is with great pleasure that Professor Piacentini and I present this closing Editorial for the Special Issue of Sports on “Maximising Triathlon Health and Performance: The State of the Art”. Thirty-four papers—of which 52% were accepted for publication—were submitted to this Special Issue. At the time of writing in December 2024, the 18 published papers had already been viewed over 106,000 times. Vleck et al.’s publication (Paper (P)3), entitled “Work, Training and Life Stress in ITU World Olympic Distance Age-Group Championship Triathletes”, has been shortlisted for the Sports Paper of the Year 2023 award. Our featured authors (listed below) include many world-renowned experts in the field. Many other subject experts, including the coach of an Olympic medallist, devoted significant time and expertise to this project as reviewers. Notably, several of the submissions (e.g., P16, Hotfiel et al., 2019) resulted from collaborations between researchers and National Federation staff. We are privileged that our contributors chose to publish their research findings, including that of a randomised control trial (P10, Grim et al., 2019), with us. We are happy to have also featured first author papers from several (then) Ph.D. students- the incoming generation of triathlon subject experts. We extend our congratulations to those authors who have since been awarded their doctorates: Dr Claudio Quagliarotti (ITA) [1], Dr Alba Cuba-Dorado (ESP) [2], Dr Joel Walsh (AUS) [3], and Dr Jørgen Melau (NOR) [4]. Dr Thibaut Ledanois (FR) [5], Dr Stuart Evans (AUS) [6], and Dr Christian Weich (GER) [7] also recently obtained their triathlon-related doctorates. João Henrique Falk Neto (CAN), who proved himself an outstanding compère of the Edmonton 2020/2021 ITU Science and Triathlon conference; Héctor Arévalo-Chico (ESP), who both conducted research with and recently accompanied members of the Spanish team to the Paris Olympic Games; and Atsushi Aoyagi (JPN), whose papers we also feature in our Special Issue—are all close to submitting their Ph.D.’s [8,9,10]. We sincerely thank Atsushi Aoyagi for kindly accepting Dr Vleck’s invitation to include his paper on the exercise intensity at which age group triathletes race in Olympic distance triathlons in this Special Issue. In common with many of the other papers in the Special Issue, Aoyagi et al.’s paper is surely destined to become regarded as a classic in the field.

The number- 10- and the submission dates, which are reasonably close to each other, of all the aforementioned doctoral theses is a testament to how scientific research related to our sport has evolved. The first triathlon paper was published just forty years ago. To our knowledge, the first doctorates on triathlons (both of which included National Squad athletes in their subject groups) were awarded to Professor Grégoire Millet (in 1999) [11], followed by myself [12]. Professor Millet was a former French National Olympic distance triathlon champion and a French National Triathlon Squad coach. In 2000, he was British Triathlon’s Performance Director for triathlon’s first Olympic Games. As such, he was an exemplar of what is an increasingly distinguishing feature of those who are involved in triathlon research. The fact that so many of our peers are actively researching *and* coaching *and/or* training for/racing triathlon (and sometimes all three at the same time), as Grégoire himself pointed out, is likely to facilitate the successful dissemination of our research findings. Almost all of the individuals whose work is featured in this Special Issue possess these same characteristics. Importantly, we were privileged to have published contributions from several researchers with a fourth, immensely valuable feature. As doctors, paramedics, physiotherapists, and/or surgeons, they have active field-, medical tent-, and/or hospital-based experience of protecting the health and safety of athletes. Dr Jørgen Melau—a paramedic and the safety director of the Norseman Xtreme triathlon, who is now the holder of a Ph.D. on physiological changes induced by cold water swimming [4]—personifies these outstanding individuals. Importantly, our authors also include those who have been (Engelhardt, Vleck) longstanding representatives on the committees or boards of either their respective continental governing bodies or World Triathlon. Thus, all of our contributors were uniquely placed to bring this collation of papers, with its stated aim of acting as a spur to the instigation and expansion of collaborative research projects that have the potential to improve applied practice in the sport, to fruition.

In 2007, Millet and I, together with David Bentley, published an invited commentary [13] on the extent to which a reciprocal relationship existed between the development of the sport of triathlon and the nature of the scientific investigation that was directly related to both it and to endurance sport in general. This Editorial provides us with a timely opportunity to revisit the questions that we first posed in the *International Journal of Sports Physiology and Performance* (IJSPP) 17 years ago. They fall under three main headings. Has science influenced the knowledge and practices in a given sport? Is sports science part of the development of the sport? Is sports science an important parameter for the emergence of new practices (i.e., training or testing methods and technological development) or coach education, and vice versa?

In Table 1, we provide selected examples of the above issues being addressed in the literature as a whole. We do the same for the three areas for which this Special Issue invited submissions, namely:

(a) Triathlon health in training and/or competition (health evaluation, event medical care, open water swimming, and heat acclimation) (Papers 7, 9, 13, 16, and 18).
(b) Training and risk factors for maladaptation (as evidenced by injury, illness, and non-functional overreaching and/or performance stagnation), including how it may change with athlete age, ability level, and event distance specialisation (Papers 3 and 5).
(c) Optimising training and race preparation- at the cutting edge (preparation for the Olympic Games, what can scientists tell coaches, what can coaches tell scientists, what do athletes want, and how technology can change the game, e.g., as regards research into both the aetiology and the prediction of maladaptation) (Papers 1, 2, 4, 6, 8, 10–12, 14–15, and 17).

Essentially, our reply to the above key questions about whether reciprocity exists between the sport and research related to it is “yes, to some extent, but more work is needed”. We highlight the fact that the event guidelines for open-water swimming have been revised on the basis of research that was commissioned and funded by this sport’s governing body, amongst others. Research that has the potential to directly improve event medical care (e.g., P10, Melau et al., 2019. Late-Presenting Swimming-Induced Pulmonary Edema: A Case Report Series From The Norseman Xtreme Triathlon) is continuously being published. Surprisingly, however (and especially given the opportunities that amateur triathlon’s unique age-group system provides for research into the effects of multi-disciplinary exercise training in ageing populations), few comprehensive studies of either triathlon training or of its long-term effects exist. The latter point is relevant to medical issues (e.g., the incidence of skin cancer; see [40]). It also applies to research into how triathlon participation might offset the effects of ageing and (given that it is a multi-disciplinary sport) might affect those at the other end of the age spectrum, e.g., motor skill development of younger athletes (of less than 8 years of age) [54,55]. There also appears to have been little investigation to date on the extent to which triathlon participation can either positively [56]) or negatively [57] impact mental health, and/or non-communicable disease [51]. It would prove useful if how triathletes actually train were better researched- both for those who take part in the sport and, probably, for sports science in general. We further recommend that more research be carried out into the extent to which current coaching accreditation courses and coaches themselves are both up to date with and implement findings from the triathlon literature. We applaud the sterling work of Professor Romauld Lepers on the decline of performance with age in masters athletes [58,59,60,61]. Lepers’ work to make the practical implications of his findings available to the wider exercising public (Lepers, 2021 [62]) sets an excellent example for us all.

We are privileged that this Special Issue was met with such an enthusiastic, collaborative response from the triathlon community worldwide. We are confident that the papers contained within it should also prove relevant to its component sports, and we extend our grateful thanks to everyone who was involved and continues to support research in our sport. We note that the analysis that I conducted in 2007 for the IJSPP commentary [13] was based on the 278 articles with triathlon or triathlete in the title that were published in PubMed between the first published paper in 1984 and the end of 2006. After four decades of research https://lida.sport-iat.de/dtu-triathlon/ (LIDA)- the database of scientific literature related to triathlon that is based on a collaboration between the Deutschen Triathlon Union (DTU) and the Institute of Applied Training Science Leipzig (IAT), now numbers over 1440 triathlon articles and over 19,000 triathlon related items. This database is freely available. In closing this Editorial, we therefore extend a special thank you to Birgit Franz, who was working on the aforementioned database when I started my triathlon-related Ph.D. over thirty years ago, and still works on it, for her significant contribution to the sport.

Finally, we wish to both acknowledge the work of Professor (Doug) Hiller, M.D., and make a related announcement. Professor Hiller was one of the first three members of the medical committee of the International Triathlon Union (ITU, now World Triathlon) in 1989. He (together with Pamela S. Douglas M.D. and Professor Mary L. O’Toole) was one of the initial leading trio of researchers to publish on triathlon and was involved with drafting the first set of medical guidelines for what would eventually become USA Triathlon. He was inducted into the Hall of Fame of the ITU in August 2019 for his lifetime contributions to this evolving Olympic sport.

The triathlon chapter in the 2010 IOC book on epidemiology of injury and illness in Olympic sports [45] concluded with these words: “It is strongly urged that a collaborative research team of race organizers, technical officials, coaches, athletes, medical support staff, and researchers working at both the grass-roots and the top end of the sport be established, for an adequate database of injury data to be compiled and used to drive continuous improvement in triathlon training and competition practice, as well as education of athletes, coaches, and both technical and medical staff”. Professor Hiller and Professor Christopher Connolly of Washington State University have now set up such a global triathlon safety database (GTSD, www.globaltrisafety.org). The GTSD is both a medical data repository and provides secure data management and analysis for triathlon organizations worldwide. To date, it is supported by the World Triathlon, Ironman Triathlon, and USA Triathlon. In addition to the LIDA database and the recently established Triathlon Research Initiative (www.triathlonresearchinitiative.com), it is likely to prove to be a critically valuable resource for the sport.

We strongly encourage all of our colleagues in the sport to make use of the GTSD and, in so doing, improve the translation of research findings into improvement in applied practice because

“Nothing is more important than the health and safety of the athlete.”

(Hiller, undated quote).

 

Sincerely,

Veronica Vleck, Ph.D.

Professor Maria Francesca Piacentini, Ph.D.

## Figures and Tables

**Table 1:** Comments on the extent of reciprocity between the development of triathlons and that of triathlon-related research.

Question	Comment	Selected Examples	
		The Literature	This Special Issue
1a. Has science influenced the knowledge and practices in a given sport?	Yes, regarding event medical care. The OWS rules for both open-water swimming and triathlon swimming were amended in 2013 in light of research that was unfortunately precipitated by the death of Fran Crippen in a 10 km event in the UAE (see Miller and Wendt, 2012 [14]). At the time of Crippen’s death, only lower and not upper water temperature limits for OWS existed. Saycell et al. later conducted research to provide a scientific rationale for lower water temperature and wetsuit rules for elite and sub-elite triathletes. They recommended a minimum water temperature of 12 °C for racing in wetsuits and of 16 °C without wetsuits. The ITU rules for racing were changed accordingly (January 2017).	Bradford et al., 2015 [15]; Saycell et al., 2018 [16].	Too early to say, but several papers have implications for applied practice, e.g., (both 2019 studies by Melau et al.) P9. Core Temperature in Triathletes during Swimming with Wetsuit in 10 °C Cold Water; and P18. Late-Presenting Swimming-Induced Pulmonary Edema: A Case Report Series from the Norseman Xtreme Triathlon; P16. Hotfiel et al., 2019. Accelerating Recovery from Exercise-Induced Muscle Injuries in Triathletes: Considerations for Olympic Distance Races; and P17. Extebarria, Mujika & Pyne, 2019. Training and Competition Readiness in Triathlon.
	Harris et al. [17] showed that the SCD rate for triathlons is higher in the swim section than it is in the cycling and running sections. It is not yet clear why. IM swimming has introduced staggered swim starts. We are not aware of any studies having yet compared the death/medical incident rates before and after this rule change—presumably there are not enough data yet available to carry out such an analysis. See https://triathlon.org/medical/ppe for details on the recommendations and regulations of WT as regards periodic health evaluation.	Di Masi et al. (2022) [18]; Harris et al., (2017) [17]; Windsor, Newman & Shephard (2020) [19].	
1b. Can it?	Yes, regarding event medical care (see the comments in the main text regarding the GTSD), but considerably more research is warranted. Several published papers to date have important implications for event medical care. Rimmer and Coniglione [20] and Felletti et al. [21] show that it may be possible, once enough quality data are collected and analysed, to both optimise the medical staff/athlete support ratios, and tailor the specificity of staff medical training, to different locations around the race course as a function of event distance, event format, athlete ability, and environmental conditions. Event medical guidelines, e.g., those of WT, include recommended staff/athlete staffing ratios, but given the paucity of research into this subject, it is difficult to determine on what basis these ratios have been set and to what extent they are valid and/or could be improved. This point applies to several other aspects of current race-related medical guidelines. For example, “the current World Triathlon heat policy that is used for Para athlete events is based on recommendations for non-disabled persons. Future studies should explore whether a specific heat policy for Para triathletes is needed, tailored in a similar manner to the exertional heat stroke policy for Para athletes” [22]. There is still no academic consensus statement on the definition and reporting of injuries and illness in triathlons.	Rimmer & Coniglione, 2012 [20]; Felletti et al., 2022 [21]; Nilssen et al., 2023, 2024 [23,24]; Johnson et al., 2023 [25]; Cho et al., 2024 [26].	
1c. Is the sport benefiting from science?	Yes. We are aware of several doctoral theses (e.g., Malcata, 2014 [27]; Ledanois, 2023 [5]) that focused on a particular country’s preparation for the Olympic Games.		Again, it is still too early to say, but several papers have implications for applied practice, e.g., P4. Alba Cuba et al., 2017 regarding talent ID, whose findings are themselves largely explained by P14. Piacentini et al., 2019’s analysis of WTS events.
1d. Is science benefiting from the sport?	Yes e.g., hyponatremia research. Triathlon studies on thermoregulation and fluid balance were instrumental in the formulation of international recommendations on preparation for endurance sports (e.g., the ACSM position stand on exercise and fluid replacement, [28]), plus work into the decline of performance with ageing. Dasa et al., 2024 [29] “questions the validity of the current metabolic limits” and “suggests a new perspective on what is physiologically achievable in world-class athletes”. However, surprising gaps exist in the literature as regards several issues that have wider implications outside the sport (e.g., the effect of triathlon participation on cardiac function, the injury and long-term health implications of multi-disciplinary cross-training across the lifespan).	Convertino et al., 1996 [28]; Noakes et al., 1985 [30]; Speedy et al., 1987 [31].	P15. Falk Neto et al., 2024. The Characteristics of Endurance Events with a Variable Pacing Profile—Time to Embrace the Concept of “Intermittent Endurance Events”?
1e. Is science influenced or stimulated by a given sport?	Yes. See the above.		
1f. Are investigations of specific interest for athletes, coaches, medical staff and administrators who are involved in that sport?	Athletes	Jeukendrup, Jentjens & Mosely, 2012 [32].	P2. Arevalo Chico et al., 2024; P17. Extebarria, Mujika & Pyne, 2019.
	Coaches	Van Schuylenburgh, Eynde & Hespel, 2004 [33]; Jacko et al., 2024 [34]; Christensen, 2025 [35].	P2. Arévalo-Chico et al., 2024; P3. Vleck et al., 2023; P14. Piacentini et al., 2019.
	Medical staff	Gailey and Hartsch, 2009 [36]; Chalmers et al., 2021 [37]; Armstrong et al., 2024 [38].	P10. Grim et al., 2019 (plantar fasciitis therapy, RCT); P18. Melau et al., 2019.
	Administrators	Wicker et al., 2012 [39]; Downs et al., 2020 [40]; Heilman et al., 2024 [41]; Bevins, 2024 [42].	P7. Seifarth et al., 2019; P8. Brown, 2019.
2a. Is sports science part of the development of the sport?	Yes, e.g., MTAR research.	Ledanois et al., 2023 [43].	Bike run transition research, e.g., P11. Olcina et al., 2019; P12. Walsh, 2019.
2b. Are the scientific topics modified by the change in rules or development stages in the sport?	Yes, see the above and Millet, Bentley & Vleck, 2007 [13] for detailed charts.		P15. Falk Neto et al., 2024.
2c. With the emergence of a sport, did it renew interest or influence the scientific investigation by providing new topics, “new borders”?	Yes regarding hyponatremia-related research. The newish Arena Games triathlon may provide a useful model for future talent identification (see Stapley et al., 2024 [44]).	See 1d.	P15. Falk Neto et al., 2024.
3a. What is the relationship between scientists, athletes, coaches, and administrators?	It exists on mostly individual bases. At a world level, it could be made more coherent and better structured. The formation of both WT coaching course accreditation and the Global Triathlon Safety Task Force (see https://education.triathlon.org/mod/page/view.php?id=10899) were very promising developments.	Nilssen et al., 2023; 2024; Johnson et al., 2023, Cho et al., 2024 (GTSD analyses of 30 years of IM data) [23,24,25,26].	P16. Hotfiel et al., 2019 is one of the multiple examples in this Special Issue of this relationship working beautifully.
3b. Are the continental or international governing bodies supporting scientific projects or congresses?	A triathlon chapter was included in Caine, Harmer, and Schiff’s *IOC Encyclopaedia of Sports Medicine Series* book on “Epidemiology of Injury in Olympic Sports”.	Vleck (2010) [45].	
	The “*Triathlon Medicine*” book was supported by WT.	Migliorini (Ed.), 2010 [46].	
	GTSD (see the main text). See also https://education.triathlon.org/mod/page/view.php?id=10899.		
	Several ITU Science of Triathlon World Congresses have taken place (Alicante 2011, Macolin 2013, Paris 2015, Edmonton 2020/2021).		
	OWS temperature limits research was funded by FINA, the IOC Medical Commission and the ITU. Saycell et al. (2018) [16] was funded by the Joint Medical Committee of the IOC, FINA, and ITU.	Bradford et al., 2015; Saycell et al., 2018 [15,16].	
3c. Are the scientists involved directly with elite or national teams or with developing junior or youth athletes?	Yes. Multiple Ph.D. theses undertaken thus far were in collaboration with National Federations and/or National Squad athletes (e.g., those of Ledanois (FR) [5], Cuba-Dorado (ESP) [2], Vleck (GBR) [12], Millet (FR) [11], Malcata (NZ) [27], Extebarria (GBR) [47] and Comotto (ITA) [48]); and several published scientists (e.g., Bottoni, Cejuela, Millet, Piacentini, and Vleck) work(ed) with National Squads.	Cuba-Dorado, 2017 [2]; Ledanois, 2023 [5].	P2. Arévalo-Chico et al., 2024; P13. Neidel et al., 2019; P14. Piacentini et al., 2019; P17. Extebarria, Mujika & Pyne, 2019; P4. Alba Cuba et al., 2021.
3d. Is sports science an important parameter for the emergence of new practices (training or testing methods, technological development) or coach education?	Yes, but its transfer into applied practice could perhaps be improved (see Quagliarotti et al., 2024 [49]; Wells et al., 2024 [50]) as could the amount of research that is undertaken on, e.g., paralympic triathletes and minority groups. See also Vleck, 2018 [51]. The “Health Triathlon Coaching program” of FFTri and the French Ministry of Sport (see Coste et al., 2020 [52] for an overview) is an inspiring example of a training program that enables triathlon coaches to work with individuals who have stable chronic diseases and in so doing, take advantage of the potential advantages of swim, cycle and run/walk training for general health.	Weich et al., 2022 [53].	P1. Procida et al. 2024; P3. Vleck et al., 2024; P8. Brown, 2019.
3e. Is a given sport only a vehicle for general scientific investigation, or are there some sport-science research initiatives specific to it?	Sports-specific initiatives exist, e.g., wetsuit research, bike-run transition research, and pacing research (including, most recently, those related to the MTAR).	Ledanois et al., 2023 [43].	P12. Walsh, 2019.

Key: ACSM, American Society of Sports Medicine; ESP, Spain; FINA, Fédération Internationale de Natation; FFTri, Fédération Française de Triathlon; FR, France; ID, identification; IM, Ironman distance triathlon; ITA, Italy; ITU, International Triathlon Union (now WT, World Triathlon); GBR, Great Britain; GTSD, Global Triathlon Safety Database; IOC, International Olympic Committee; MTAR, triathlon Mixed Team Athlete Relay; NZ, New Zealand; OD, Olympic Distance triathlon; OWS, open water swimming; P, paper (see the numbered list of contributors and papers); Qu, Question; re, regarding; RCT, randomised control trial; SCD, sudden cardiac death; UAE, United Arab Emirates; WTS, World Triathlon Series.
